# Sunitinib induces genomic instability of renal carcinoma cells through affecting the interaction of LC3-II and PARP-1

**DOI:** 10.1038/cddis.2017.387

**Published:** 2017-08-10

**Authors:** Siyuan Yan, Ling Liu, Fengxia Ren, Quan Gao, Shanshan Xu, Bolin Hou, Yange Wang, Xuejun Jiang, Yongsheng Che

**Affiliations:** 1 State Key Laboratory of Mycology, Institute of Microbiology, Chinese Academy of Sciences, Beijing, China; 2 University of Chinese Academy of Sciences, Beijing, China; 3 State Key Laboratory of Toxicology and Medical Countermeasures, Beijing Institute of Pharmacology and Toxicology, Beijing, China

## Abstract

Deficiency of autophagy has been linked to increase in nuclear instability, but the role of autophagy in regulating the formation and elimination of micronuclei, a diagnostic marker for genomic instability, is limited in mammalian cells. Utilizing immunostaining and subcellular fractionation, we found that either LC3-II or the phosphorylated Ulk1 localized in nuclei, and immunoprecipitation results showed that both LC3 and Unc-51-like kinase 1 (Ulk1) interacted with *γ*-H2AX, a marker for the DNA double-strand breaks (DSB). Sunitinib, a multi-targeted receptor tyrosine kinase inhibitor, was found to enhance the autophagic flux concurring with increase in the frequency of micronuclei accrued upon inhibition of autophagy, and similar results were also obtained in the rasfonin-treated cells. Moreover, the punctate LC3 staining colocalized with micronuclei. Unexpectedly, deprivation of SQSTM1/p62 alone accumulated micronuclei, which was not further increased upon challenge with ST. Rad51 is a protein central to repairing DSB by homologous recombination and treatment with ST or rasfonin decreased its expression. In several cell lines, p62 appeared in the immunoprecipites of Rad51, whereas LC3, Ulk1 and p62 interacted with PARP-1, another protein involved in DNA repair and genomic stability. In addition, knockdown of either Rad51 or PARP-1 completely inhibited the ST-induced autophagic flux. Taken together, the data presented here demonstrated that both LC3-II and the phosphorylated Ulk1 localized in nuclei and interacted with the proteins essential for nuclear stability, thereby revealing a more intimate relationship between autophagy and genomic stability.

Micronuclei are small DNA-containing membrane-enveloped structures separated from the primary nuclei of the cell, containing chromosomal fragments and/or whole chromosomes based on different types of formation.^1, 2^ Accumulating evidence has linked the frequency of micronuclei to cell types, which reflected nuclear stability.^2, 3^ As micronuclei are usually destructed by cytoplasmic nucleases, they are believed to be removed through autophagy.^4, 5^ Autophagy, one of the two major pathways degrading the cellular components in eukaryotic cells, mainly controls the turnover of long-lived proteins and organelles.^6^ Unc-51-like kinase 1 (Ulk1) and LC3 are two central components in autophagy, whereas Ulk1 participates in the induction process,^7^ the phosphatidylethanolamine-conjugated form of LC3 (Atg8-PE/LC3-II) is the only reliable marker protein related to completed autophagosomes.^8^ Accumulation of DNA damage has been found in autophagy-deficient mammary tumor cells,^9^ and either nuclear components or nuclear lamina were degraded by the autophagic process.^10, 11^ In senescent cells, autophagy participates in proteolytic processing of histones, the basic structural proteins of eukaryotic chromosomes.^12^ These reports indicate that autophagy has an important role in the DNA damage repairing process.

Non-homologous end joining (NHEJ) and homologous recombination (HR) are the two principal mechanisms to repair DNA double-strand breaks (DSB) in mammalian cells.^13, 14^ And *γ*-H2AX (phosphorylated histone H2AX Ser139) is commonly used as a marker for DSB.^15^ Central to DSB repair by HR is Rad51, which is detected in multiple discrete subnuclear structures (foci) and promotes strand invasion and homologous pairing between the two DNA duplexes.^16, 17^ Many signaling molecules, such as Akt and extracellular regulated protein kinases 1/2 (ERK1/2), affected the expression of Rad51, a demonstrated negative regulator of autophagic process.^18, 19, 20^ In addition, inhibition of autophagy enhanced radiosensitivity of nasopharyngeal carcinoma via reducing the expression of Rad51.^21^ Recently, autophagy has been demonstrated to regulate chromatin ubiquitination in DNA damage response (DDR) through elimination of p62.^17, 22^ Besides Rad51, the DNA-binding enzyme poly(ADP-ribose) polymerase 1 (PARP-1) is also involved in modulating the activity of the DNA repair systems,^23^ has a primary role in the process of poly(ADP-ribosyl)ation, and is responsible for the major poly(ADP-ribosyl)ation activity observed during DDR. PARP-1 has been demonstrated to link to the ataxia-telangiectasia mutated protein (ATM), a key signal transducer having a critical role in DDR.^24^ Its over activation induces mitochondrial transition and damage, leading to cell death,^23^ and its cleavage facilitates cellular disassembly and serves as a marker for cells undergoing the caspase-dependent apoptosis.

Sunitinib (ST), approved by FDA to treat renal cell carcinoma (RCC),^25^ inhibits the activity of PDGFRs, c-KIT, FLT-3 and the VEGFRs.^26^ It not only induces cell viability loss and cell senescence, but also causes G1-S cell cycle arrest and DDR in OS-RC-2 cells.^27^ In addition, it was reported to stimulate incomplete autophagic flux in renal and bladder cancer cells,^28, 29^ and to induce autophagy in cardiac cells and PC12 cells.^30, 31^ Rasfonin is a natural product isolated from the fermentation cultures of the fungus *Talaromyces* sp. 3656-A1, and was named after its biological activity against the small G-protein Ras.^32^ In our previous study, it was found to induce both apoptosis and autophagy.^33^

In this study, we have demonstrated that both ST and rasfonin increased the level of *γ*-H2AX, reduced the expression of Rad51, and stimulated the formation of micronuclei. Moreover, we found that LC3-II and pUlk1 localized in nuclei, and colocalized with *γ*-H2AX in micronuclei. Knockdown of LC3 or Ulk1 increased the frequency of micronuclei, which was further accumulated upon challenge with ST. However, ST failed to further increase the formation of micronuclei in the p62-depleted cells. Immunoprecipitation results showed that LC3 interacted with *γ*-H2AX, Rad51 and PARP-1. Although both Rad51 and PARP-1 can bind to p62 and deprivation of either one completely inhibited the ST-induced autophagy, they affect the micronuclei formation in different manners.

## Results

### ST inhibits cell viability and induces autophagy in renal cancer cells

**Figure 1:**
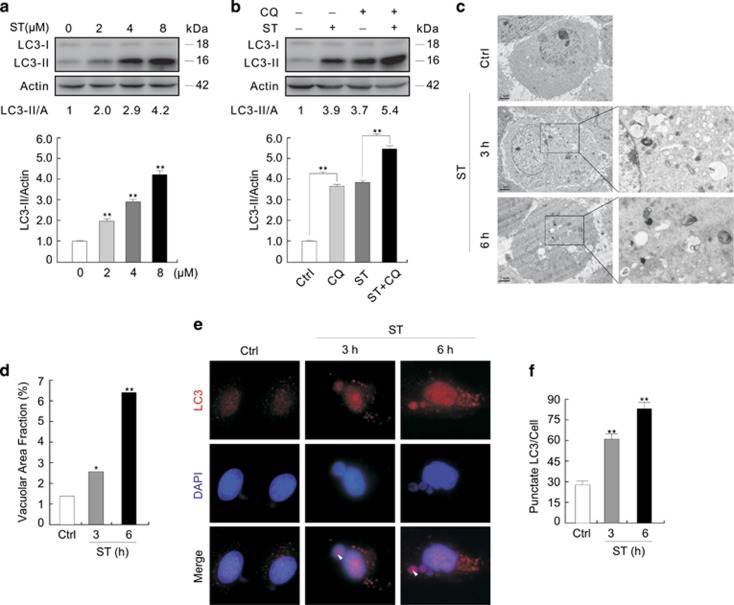
ST stimulates autophagy in renal cancer cells. (**a** and **b**) Following treatment with ST (8 *μ*M; unless otherwise indicated) for 3 h in the presence or absence of 10 *μ*M CQ, 786-O cells were lysed and subjected to immunoblotting with the antibodies indicated. Images from three independent experiments were analyzed by Image J, and mean±S.D. were shown in histograms along with the blots. (**c**) Electron microscopy was performed for 786-O cells following treatment with ST for 3 and 6 h. The data of the area ratio were non-normally distributed, and presented as the mean of at least 15 cells counted for each group, the data were analyzed by Friedman test (**d**). (**e**) Immunofluorescence using the antibody of LC3 was performed for 786-O cells following treatment with ST in the presence or absence of CQ for up to 6 h (arrowheads indicated the LC3 dots in nuclei, and arrows indicated micronuclei). The numbers of the punctate LC3 in each cell were counted, and at least 50 cells were included for each group (**f**). Data representing the mean±S.D. were shown in graph. *P<0.05 *versus* control; **P<0.01 *versus* control. All data were acquired from at least three independent experiments

ST reduced the viability of 786-O cells in time- and dose-dependent manners (Supplementary Figure 1A), and its cytotoxicity was further confirmed by colony growth assay (Supplementary Figure 1B). Meanwhile, high doses of ST stimulated the cleavage of PARP-1 (Supplementary Figure 1C),^34^ and flow cytometry analysis revealed its obvious induction of apoptotic cell death (Supplementary Figure 1D). ST has been reported to induce autophagy in a number of cell lines,^30, 31^ and we also found that it increased the level of LC3-II and stimulated the autophagic flux, as chloroquine (CQ) further accumulated LC3-II in the ST-treated 786-O cells (Figures 1a and b). Similar results were also obtained in ACHN cells (Supplementary Figure 2A). Under electron microscopy, it caused obvious accumulation of membrane vacuoles compared with the control (Figures 1c and d; Supplementary Figure 2B), and immunostaining results revealed significant increase in the punctate staining of LC3 in 786-O and ACHN cells (Figures 1e and f; Supplementary Figure 2C). Despite of the increased LC3 staining in the ST-treated cells, the presence of punctate LC3 staining was found in both nuclei and micronuclei (Figure 1e), indicating that LC3-II may localize in nuclei.

### Nuclear localization of LC3-II and the phosphorylated Ulk1

**Figure 2:**
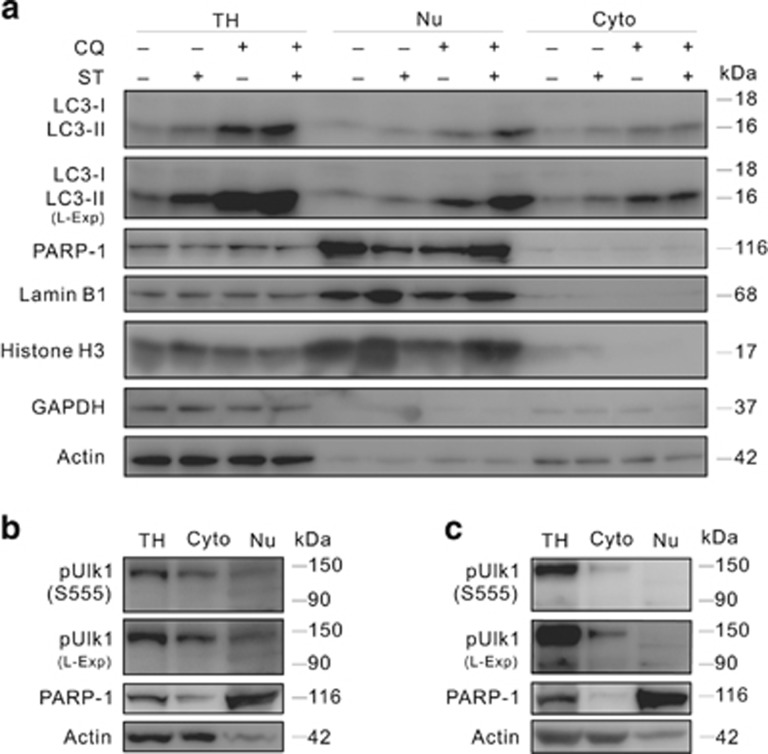
LC3-II localizes in nucleus. (**a**) The total homogenate (TH), nuclear fractions (Nu) and cytoplasm fraction (Cyto) were extracted from 786-O cells after treated with the indicated compounds for 3 h, and analyzed by immunoblotting with the antibodies indicated (L-Exp: long expose). (**b** and **c**) TH, Nu and Cyto were extracted from HEK293T or 786-O cells, resolved by electrophoresis, and probed by immunoblotting with the indicated antibodies. Similarly experiments were performed for at least three times

Deacetylation of nuclear LC3 has been reported to drive autophagy,^35^ and LC3A-II, not LC3B-II (thereafter called LC3-II), could localize in nuclei.^36^ Although LC3-II is usually considered as a cytoplasmic protein, it may localize in nuclei as suggested in recent study.^11^ To confirm its nuclear localization, subcellular fractionation was performed using PARP-1 as the marker of nuclei,^37^ and LC3-II was found in the nuclear fraction and its level was further increased in the presence of ST (Figure 2a). In addition, either Lamin B1 or histone H3, two often used nuclear markers,^11, 38^ was also detected (Figure 2a). In contrast, glyceraldehyde phosphate dehydrogenase (GAPDH) did not appear in nuclear fraction (Figure 2a). Consistent with the results that Ulk1 could exist in nuclei and interact with PARP-1,^39^ we also observed nuclear localization of the phosphorylated Ulk1 Ser555 (pUlk1) in HEK293T cells (Figure 2b). Although the band for pUlk1 of normal molecular weight (NMW) was not found in the nuclear fraction of 786-O cells, a band for that of relatively lower MW (LMW) was observed in nuclei (Figure 2c), suggesting the cleavage of Ulk1 under certain circumstances. Actually, the LMW form of pUlk1 was also observed in HeLa and K562 cells (Supplementary Figures 3A and B). Moreover, the bands for both NMW and LMW Ulk1 decreased in the Ulk1-depleted HEK293T and HeLa cells (Supplementary Figure 3C), suggesting that the LMW one is specific for Ulk1. Although ST increased the nuclear-localized pUlk1 of NMW, rasfonin decreased its nuclear localization in HEK293T cells (Supplementary Figure 3D). However, the nuclear-localized pUlk1 of LMW appeared to be increased in both types of the treated cells (Supplementary Figure 3D). An online software, 'EMBOSS: sigcleave' (http://emboss.bioinformatics.nl/cgi-bin/emboss/sigcleave), was used to predict the cleavage sites of the proteins, and two candidate cutting sequences were predicted in Ulk1 (Supplementary Figure 3E). Considering their MWs, the sequence between serine-381 and alanine-393 could be the site of cleavage. Interestingly, LC3-II and pUlk1 were also found in the insoluble nuclear participates (Nup; Supplementary Figure 3D), which was supposed to be chromatin.^40^

**Figure 3:**
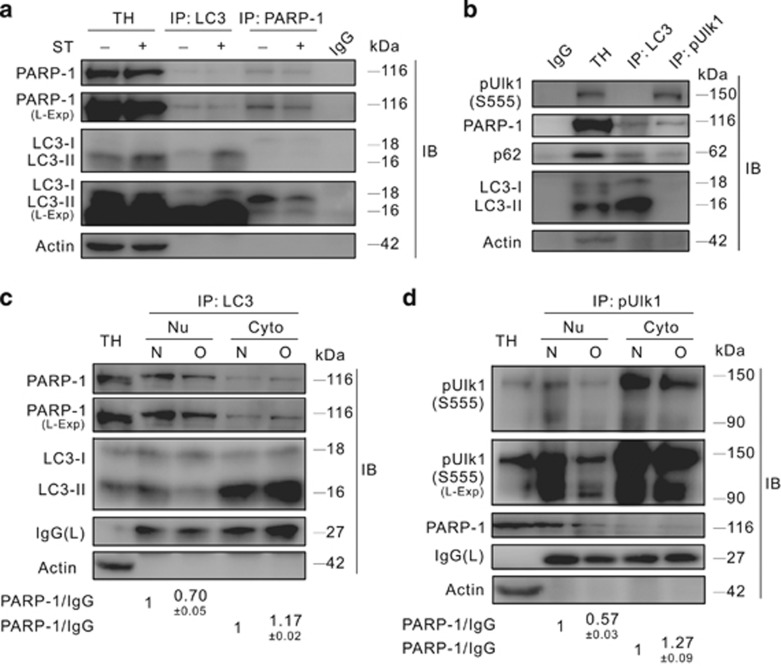
Both LC3 and Ulk1 interact with PARP-1. (**a**) 786-O cells were treated with or without ST for 3 h, cells were lysed, and precipitated using the indicated antibodies. The immunoprecipitates were resolved by electrophoresis and probed by immunoblotting with the indicated antibodies. (**b**) Immunoprecipitation was performed for the lysate extracted from 786-O cells using either the antibody of LC3 or pUlk1. IgG: the negative control antibody. (**c** and **d**) After treated with or without fresh medium for 2 h, immunoprecipitation was performed for the Cyto and Nu fractions extracted from HEK293T cells using the antibody of LC3 and pUlk1, respectively. IgG (L): the light chain of IgG. The immunoprecipitates were resolved by electrophoresis and probed by immunoblotting with the indicated antibodies. At least three independent experiments were performed

PARP-1 is a DNA-binding enzyme and an often used nuclear marker.^23, 37^ To further confirm the nuclear localization of LC3-II, immunoprecipitation was performed using the antibody of either LC3 or PARP-1, and LC3-II was found in the immunoprecipitates of PARP-1 (Figure 3a), whereas PARP-1 appeared in the immunoprecipitates of both LC3 and pUlk1 (Figures 3a and b). LC3 was found to interact with PARP-1 in both the nuclear and cytoplasmic lysates of HEK293T cells, and the interaction in nucleus was much stronger than that in cytoplasm, although much more LC3-II was detected in the cytoplasm (Figure 3c). In the immunoprecipitates of pUlk1, relatively larger amount of PARP-1 was found in the Nu fraction than the cytoplasm one extracted from HEK293T cells (Figure 3d). Although LC3 binds to less PARP-1 in cytoplasm when cells were cultured in fresh medium (N) compared with the old one (O), their interaction was enhanced in nuclei under the condition (Figure 3c). Similar to the interaction between LC3 and PARP-1, the binding of pUlk1 to PARP-1 in nuclei was increased in fresh medium (Figure 3d).

### LC3 interacts with *γ*-H2AX and Rad51

**Figure 4:**
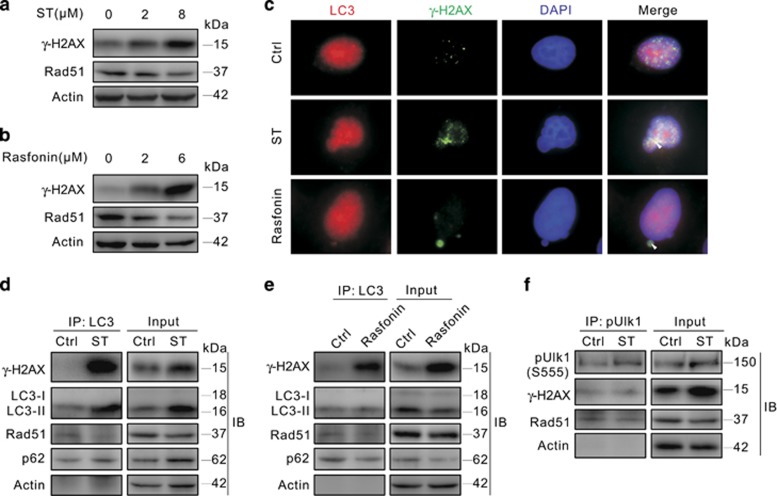
LC3 interacts with *γ*-H2AX and Rad51. (**a** and **b**) 786-O cells were treated with ST (0–8 *μ*M) or rasfonin (0–6 *μ*M) for 3 h, cells were lysed and subjected to immunoblotting with the antibodies indicated. (**c**) 786-O cells were treated with ST or rasfonin for 3 h, and the images were obtained using fluorescence microscopy following staining with the antibodies of LC3 and *γ*-H2AX. (**d**–**f**) 786-O cells were incubated with ST or rasfonin for 3 h and lysed, and LC3s or pULK1s were precipitated using the antibody against LC3 or pULK1 (Ser555). The immunoprecipitates were resolved by electrophoresis and probed by immunoblotting with the indicated antibodies. All data were acquired from three independent experiments

DNA damage elements can elevate the level of *γ*-H2AX.^15^ Both ST and rasfonin remarkably increased the protein level of *γ*-H2AX, but failed to augment the level of Rad51 (Figures 4a and b).^41^ As the formation of Rad51 foci, not the protein level of Rad51, was increased upon DNA damage,^42^ we could not completely exclude the possibility that either ST or rasfonin induced the HR. Both ST and rasfonin increased the localization of LC3 and *γ*-H2AX in micronuclei (Figure 4c), and immunoprecipitation assay revealed that LC3 interacted with *γ*-H2AX and the interaction was enhanced by either ST or rasfonin (Figures 4d and e). As the positive control, SQSTM1/p62 (p62), a selective substrate of autophagy and binding partner of LC3,^43^ was found in the immunoprecipitates of LC3 (Figures 4d and e). In addition, Rad51 interacted with LC3, but ST or rasfonin decreased the binding between LC3 and Rad51 (Figures 4d and e). Furthermore, both *γ*-H2AX and Rad51 were found in the immunoprecipitates of pUlk1, and the binding between pUlk1 and Rad51 was reduced by ST (Figure 4f).

### Deprivation of LC3 and Ulk1 increases the frequency of micronuclei

**Figure 5:**
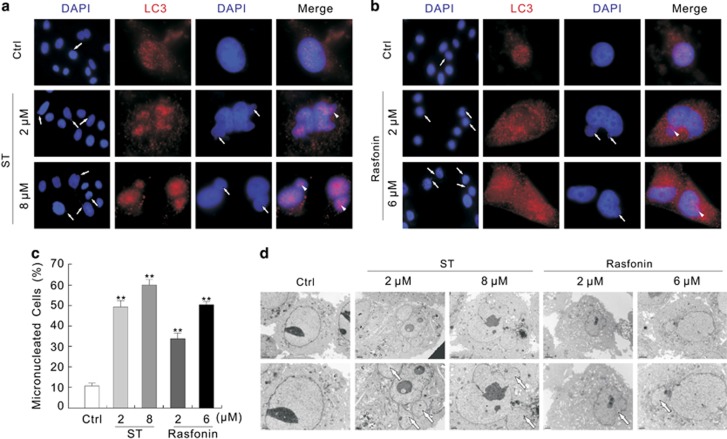
ST and rasfonin increase the formation of micronuclei. (**a** and **b**) 786-O cells were treated with ST (0–8 *μ*M) or rasfonin (0–6 *μ*M) for 3 h, and the images were obtained by fluorescence microscopy after labeling the antibody of LC3 and DAPI with both 400 and 1000 magnification. The percentages of micronuclei were analyzed and shown in (**c**), and the data were presented as mean±S.D. in graphs, **P<0.01 *versus* control. (**d**) Electron microscopy was performed for 786-O cells following treatment with ST or rasfonin for 3 h. Representative images were presented, the arrows indicated micronuclei, and arrowheads showed the LC3 localized in micronuclei. Similarly experiments were carried out for three times

As the nuclear buds and nucleoplasmic bridges can also be used as the biomarkers of genotoxic events in addition to micronuclei,^2^ all three types of genotoxic biomarkers were counted and labeled as micronuclei for the convenience of presentation (Supplementary Figure 4A). Either ST or rasfonin were found to significantly increase the formation of micronuclei in a dose-dependent manner (10% and 30–60% in untreated and treated groups, respectively; Figures 5a–c) as confirmed by electron microscopy (Figure 5d), and both caused accumulation of micronuclei in ACHN, HeLa and HepG2 cells (Supplementary Figures 4B and C), indicating that either one can be used as a genotoxic agent.

**Figure 6:**
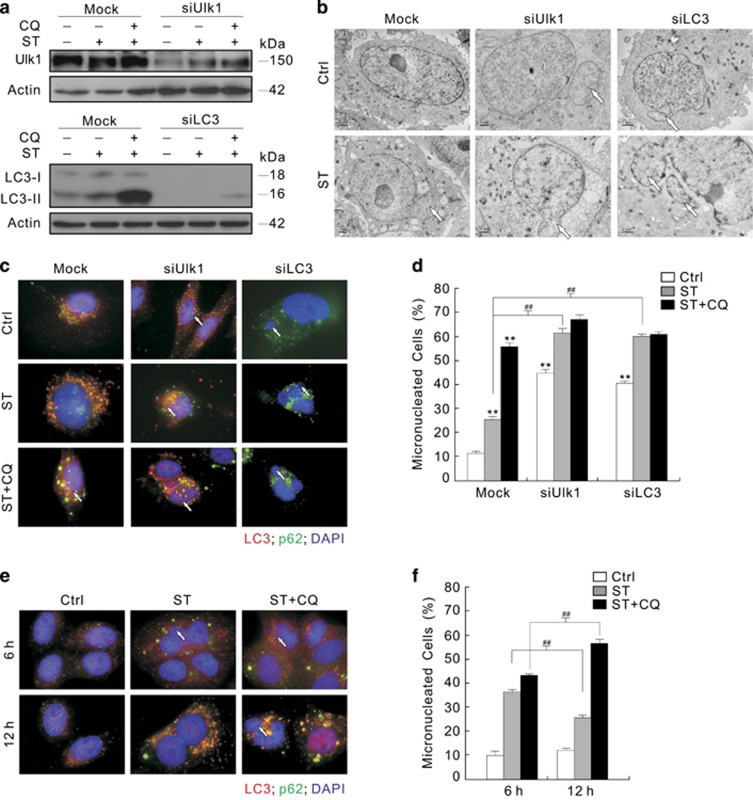
Inhibition of autophagy increases the percentage of micronuclei**.** (**a**–**d**) After transfection with siRNA target Ulk1 or LC3 for 48 h, 786-O cells were treated with ST or a combination with CQ for 12 h. Cell lysates were analyzed by immunoblotting with the indicated antibodies (**a**), electron microscopy images were shown in (**b**) and the images were obtained using fluorescence microscopy after labeling the antibodies of LC3 and p62 (**c**). Representative images were presented and the white arrows indicated micronuclei. Histogram graph data of micronuclei in (**c**) representing the mean±S.D. were shown in (**d**). (**e** and **f**) 786-O cells were treated with ST or a combination with CQ for up to 12 h, images were obtained using fluorescence microscopy after labeling the antibodies of LC3 and p62, and the percentages of micronuclei were calculated and presented as histogram graph. **P (^##^*P*)<0.01 *versus* control. The data represent three independent experiments

To examine the role of autophagy in the regulation of micronuclei, Ulk1 or LC3 was silenced by targeted siRNA (Figure 6a), and deprivation of either one accumulated micronuclei (Figures 6b−d), which was also confirmed using another siRNA targeted to Ulk1 or LC3 (Supplementary Figures 5A and B). Meanwhile, ST was found to further increase the percentage of micronuclei at the 12-h time point (Figures 6b−d). Treatment with CQ alone slightly increased the frequency of micronuclei (Supplementary Figure 5C), but further increased the percentage of micronuclei in the ST-treated Mock cells (Figures 6c and d; Supplementary Figure 5D). However, CQ failed to significantly augment the frequency of micronuclei in either the Ulk1- or LC3-silenced cells (Figures 6c and d; Supplementary Figures 5E and F). As autophagy was reported to participate in removal of micronuclei and to contribute to genomic stability,^4^ its role in the ST-induced formation and degradation of micronuclei was explored. We found that ST increased the formation of micronuclei in a time-dependent manner, but prolonged exposure to ST decreased the percentage of micronuclei, from ~40% at 6 h to ~30% at 12 h (Figures 6e and f; Supplementary Figures 6A and B), although the autophagic process was stimulated by ST at all the time points detected (Supplementary Figure 6C). Treatment with CQ maintains the percentage of micronuclei at a relatively high level in the ST-treated cells at both the 6 and 12-h time points (Figures 6e and f; Supplementary Figures 6A and B), suggesting that autophagy is involved in removal of micronuclei.

### Deprivation of p62 differentially affects the basal and ST-induced formation of micronuclei

**Figure 7:**
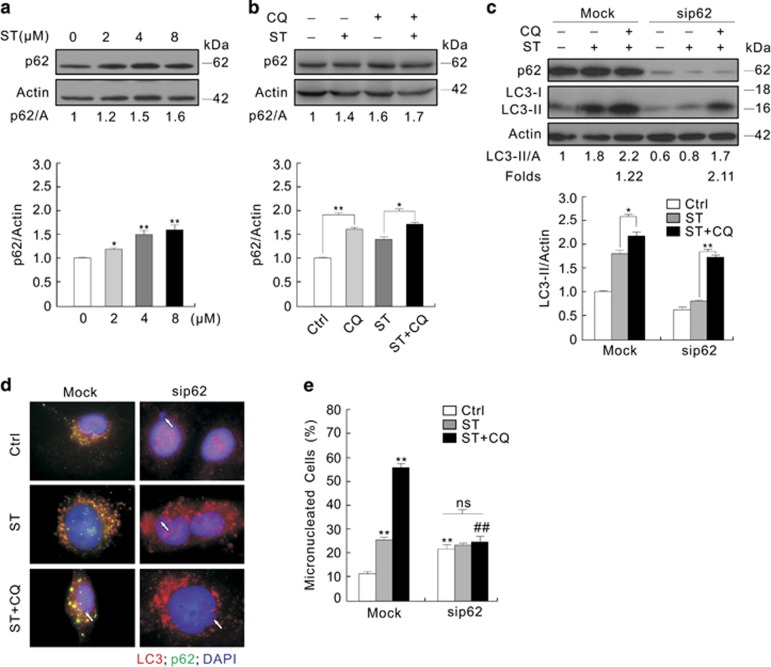
Knockdown of p62 increases the frequency of micronuclei. (**a** and **b**) Following treatment with ST for 3 h in the presence or absence of CQ, 786-O cells were lysed and subjected to immunoblotting with the antibodies indicated. (**c**–**e**) 786-O cells were transfected with siRNA target p62 for 48 h. Following treatment with ST with or without CQ for 3 h, cell lysate were prepared, immunoblotting was performed with the antibodies indicated (**c**), and treated with the indicated compounds for 12 h; images were obtained using fluorescence microscopy after labeling the antibodies of LC3 and p62 (white arrows indicated micronuclei). The histogram graph data representing the mean±S.D. were shown in (**e**). **P<0.01 *versus* control, and *P<0.05 *versus* control; ^##^*P*<0.01 *versus* the Mock group, ns was short for insignificance. At least three independent experiments were performed

As ST increased the colocalization of the punctate LC3 staining and p62, and induced much larger dots of p62, it may promote autophagy with increased expression of p62 (Figure 6e; Supplementary Figures 6A and B). In agreement with our aforementioned assumption, ST increased the expression of p62 (Figure 7a and Supplementary Figure 7A), and CQ further accrued p62 in the ST-treated cells (Figure 7b). Using quantitative real-time PCR (qPCR), we found that ST transcriptionally increased the expression of p62 (Supplementary Figure 7B), and increased the expression of Beclin 1 (Supplementary Figure 7C), suggesting that ST activated autophagy under the circumstance. As p62 was required for autophagic process,^44^ we also examined the ST-induced autophagy in the p62-depleted renal cancer cells. ST and CQ accumulated more LC3-II in the p62-depleted cells compared with the Mock controls (Figure 7c), indicating that the loss of p62 does not inhibit the ST-dependent autophagic flux. Unexpectedly, we found that silencing of p62 increased the frequency of micronuclei compared with the Mock control, but ST or its combination with CQ failed to further accumulate micronuclei in the p62-deprived cells (Figures 7d and e; Supplementary Figure 7D), indicating that the ST-induced formation of micronuclei is p62 dependent. As knockdown of either LC3 or Ulk1 increased the expression of p62 (Supplementary Figure 7E), we simultaneously silenced LC3 and p62, resulting in accumulated micronuclei, which was not further increased upon treatment with ST (Supplementary Figures 7F and G).

### Rad51 interacts with p62 and its deprivation inhibits the ST-induced autophagy

**Figure 8:**
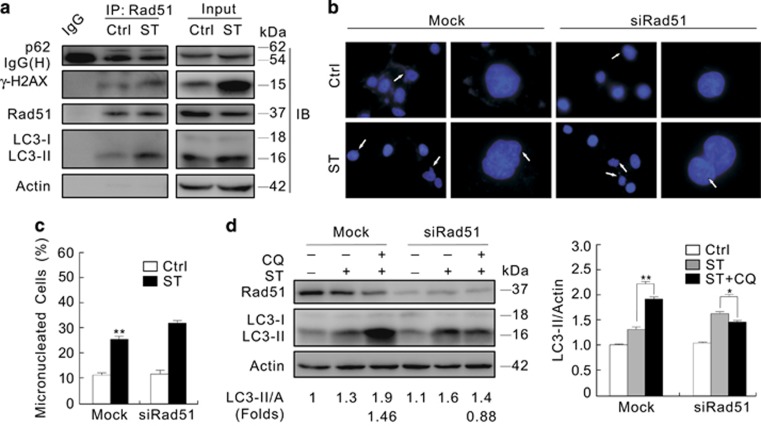
Deprivation of Rad51 completely inhibits the ST-induced autophagic flux and slightly affects the formation of basal micronuclei. (**a**) 786-O cells were incubated with ST for 3 h, lysed, and the lysates were precipitated using the antibody against Rad51 or IgG as the negative control. The immunoprecipitates were resolved by electrophoresis and probed by immunoblotting with the indicated antibodies. IgG (H): the heavy chain of IgG. (**b**–**d**) 786-O cells were transfected with siRNA target Rad51 for 48 h. Following treatment with ST for 12 h, the images were obtained using fluorescence microscopy (**b**). The histogram graph data of (**b**) representing the mean±S.D. were shown in (**c**). Cell lysate were prepared and immunoblotting was performed with the antibodies indicated for 3 h (**d**). **P<0.01 *versus* control, and *P<0.05 *versus* control. Similarly experiments were carried out for at least three times

Consistently, LC3 was pulled down in 786-O cells using the antibody of Rad51 (Figure 8a). Although p62 was found to interact with Rad51, it was not detected in the negative IgG control lane, and actin was not found either in the immunoprecipitates of Rad51 (Figure 8a). Contrarily, *γ*-H2AX was not detected in the immunoprecipitates of p62 (Supplementary Figure 8A). Although PARP-1 was also observed in the immunoprecipitates of p62 in both 786-O and HEK293T cells (Supplementary Figures 8A and B), it was not detected in the GFP immunoprecipitates (Supplementary Figure 8B). Moreover, Rad51 was readily detected in HEK293T cells using the antibody of p62 in immunoprecipitation (Supplementary Figure 8C). Similar to treatment with ST, rasfonin also decreased the binding between p62 and Rad51 (Figure 8a and Supplementary Figure 8C), and the interaction between them was also observed in HeLa cells, (Supplementary Figure 8D).

Unlike deprivation of p62, the loss of Rad51 did not markedly increase the frequency of micronuclei in 786-O cells, whereas ST still stimulated the formation of micronuclei in the Rad51-depleted cells (Figures 8b and c). However, depletion of Rad51 alone accumulated significant amount of micronuclei in HeLa cells (Supplementary Figures 8E and F), suggesting that Rad51 may regulate nuclear stability in a cell type-dependent manner. In contrast, the loss of Rad51 completely inhibited the ST-induced autophagic flux as CQ failed to accumulate LC3-II in the cells (Figure 8d).

### PARP-1 interacts with p62 and its loss completely inhibits the ST-induced autophagy

**Figure 9:**
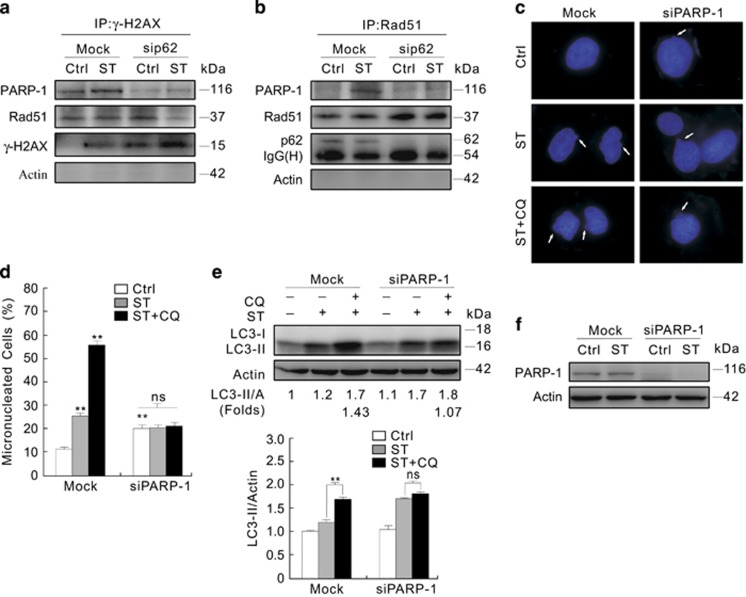
Depletion of PARP-1 alone significantly increases the frequency of micronuclei and blocks the ST-dependent autophagy. (**a** and **b**) After transfection with p62 siRNA for 48 h, 786-O cells were incubated with ST for 3 h, lysed, and the lysates were precipitated using the antibody against *γ*-H2AX or Rad51. The immunoprecipitates were resolved by electrophoresis and probed by immunoblotting with the indicated antibodies. (**c**–**f**) 786-O cells were transfected with siRNA target PARP-1 for 48 h. (**c**) Following treatment with ST with or without CQ for 12 h, images were obtained using fluorescence microscopy, and the histogram graph data representing the mean±S.D. were shown in (**d**), **P<0.01 *versus* control. (**e** and **f**) Cell lysates were prepared and immunoblotting was performed with the indicated antibodies for 3 h. The data represent three independent experiments. **P<0.01, and ns was short for insignificance

Immunoprecipitation was performed to investigate the accumulation of micronuclei in the p62-depleted 786-O cells. Deprivation of p62 remarkably reduced the ST-induced interactions between *γ*-H2AX and Rad51, and PARP-1 (Figure 9a), and that between Rad51 and PARP-1 was also attenuated after treatment (Figure 9b). However, silencing of p62 increased the amount of Rad51 in the immunoprecipitates of *γ*-H2AX, and slightly affected the interaction between Rad51 and PARP-1 in untreated cells (Figures 9a and b). Considering that the frequency of micronuclei in the p62-silenced cells, and the recruitment of repair factors by *γ*-H2AX during DSB,^45^ p62 participates in regulating the complexes for DNA repairing and regulates nuclear stability likely through PARP-1 in 786-O cells, and the lack of DNA repair resulted from ST treatment in the p62-silenced cells may lead to failure in increase of micronuclei. Given that PARP-1 is related to genomic stability and DNA repairing,^23, 24^ whether PARP-1 had a role in the ST-induced DNA damage and autophagy was explored. Similar to p62 silencing, ablation of PARP-1 increased the percentage of micronuclei compared with the Mock control (Figures 9c and d; Supplementary Figure 9A), and double depletion of Rad51 and PARP-1 significantly accumulated micronuclei in 786-O cells (Supplementary Figure 9B). Moreover, either ST or its combination with CQ failed to augment the frequency of micronuclei in the PARP-1-depleted cells (Figures 9c and d; Supplementary Figure 9A), and depletion of PARP-1 completely inhibited the ST-dependent autophagic flux (Figures 9e and f).

These data revealed a closer relationship between nuclear stability and autophagy, suggesting that PARP-1 is likely to have a role in connecting the autophagic pathway to the nuclear stability, and p62 may regulate nuclear stability by binding to PARP-1 and by affecting the interaction between PARP-1 and *γ*-H2AX (Supplementary Figure 9C).

## Discussion

We have demonstrated that either LC3-II or pUlk1 showed the characteristics of nuclear localization, and both LC3 and pUlk1 can interact with *γ*-H2AX, Rad51 or PARP-1, all involved in maintaining genomic stability. Notably, both LC3-I and LC3-II were found in the immunoprecipitates of PARP-1, and Rad51 was found to interact with LC3-II. We thus propose that the nuclear-localized LC3 or pUlk1 likely functions uniquely to link genomic stability to autophagy, two important areas of biology.

LC3-II has been implied to localize in nuclei,^11, 36^ which has now been confirmed in our study. The autophagic initiator pUlk1 (Ser555) was also found in the nuclear lysates in significant amount. Interestingly, LC3-II was also found in the insoluble nuclear precipitates containing mainly genomic DNA,^40^ we therefore assumed that LC3-II may have much broader roles than previously recognized.^8, 46^ Our data showed that PARP-1 bound to LC3-I and LC3-II, and both LC3 and pUlk1 interacted with Rad51. As a result, the autophagic proteins could regulate nuclear stability through either Rad51 or PARP-1. In addition, we observed a band for relatively LMW pUlk1 in the nuclear fraction. Actually, cleavage of the autophagy-targeted proteins happened not solely to Ulk1. As the results from previous studies have shown that both Atg5 and Beclin 1 can be cleaved,^47, 48^ and caspase 8 was found to cleave Beclin 1, leading to inhibition of autophagy,^49, 50^ it is important and necessary to explore the function of the LMW Ulk1 in future study.

As ST failed to accumulate micronuclei in either the p62- or p62/LC3-depleted cells, the ST-induced formation of micronuclei is obviously p62 dependent, and the basal and induced accumulations of micronuclei are likely differentially regulated because of the fact that knockdown of p62 alone increased the frequency of micronuclei. Although p62 is often used as a substrate of autophagy, mounting evidence has indicated that it has more active roles in regulating autophagy.^44^^51^ Consequently, p62 may regulate autophagy in a stimulus- or/and cell type-dependent manner. Besides LC3 and Ulk1, p62 was also found to interact with both Rad51 and PARP-1 in this study, suggesting that it may regulate nuclear stability through either one or both. As p62 is a protein connecting the ubiquitin system and the autophagic machinery,^17^ and depletion of Rad51 or PARP-1 completely inhibited the ST-induced autophagic flux, it is very likely that both the autophagic and ubiquitin systems coordinate to regulate nuclear stability. Together with the finding that ST failed to augment micronuclei in the PARP-1-deprived cells, we speculated that p62 may regulate the formation of micronuclei through PARP-1, which has been reported to vigorously participate in the regulation of autophagy.^52, 53^ Different from silencing of Rad51, ablation of PARP-1 resulted in nuclear instability in 786-O cells, indicating that PARP-1 is more important than Rad51 to maintain the basal genomic stability at least in this cell line. Interestingly, observation of interaction between LC3 and PARP-1 in nuclear lysates and co-immunoprecipitation of Rad51 with LC3-II implied that the nuclear localization of LC3-II may participate in maintaining genomic stability.

Although commonly used as a marker for DSB, *γ*-H2AX indeed actively participates in the DNA repairing process,^45, 54^ and it was thought to function as an adaptor for recruiting modifying factors of chromatin remodeling.^55^ Here, it was found to interact with either PARP-1 or Rad51, which were disrupted by the loss of p62. Therefore, these proteins presumably formed different regulatory complexes to participate in maintaining genomic stability or other physiological processes. Although either ST or rasfonin elevated the level of *γ*-H2AX, both reduced the expression of Rad51 essential for HR repair, implying that ST and rasfonin induced acute DNA damage leading to cell death; on the other hand, they may cause chromatin remodeling and induce the error-prone DNA repair mechanisms, NHEJ. Given that depletion of either PARP-1 or Rad51 completely inhibited the ST-dependent autophagic flux, we propose that a balance or a switch may exist in these proteins to regulate autophagy and genomic stability. In fact, we observed that the interaction between LC3 and Rad51 was regulated by culturing condition, and the binding between LC3 and Rad51 was enhanced by fresh medium in nuclei concurring with decreased interaction in cytoplasm.

In summary, our data revealed more intimate and complicated relationship between autophagy and nuclear stability. The nuclear localization of LC3-II/pUlk1 and their interactions with PARP-1 resulted in their direct functions in this cellular organelle. Future work in this direction will provide more clues for better understanding of the mechanisms for necleophagy and non-selected autophagy.

## Materials and methods

### Chemicals and antibodies

ST (S126061) was acquired from Aladdin (Seattle, WA, USA). CQ diphosphate salt (C6628) and polyclonal antibodies against LC3B (L7543) were purchased form Sigma-Aldrich (St. Louis, MO, USA). The antibodies of PARP-1 (9542), Rad51 (8875), Beclin 1 (4122), phospho-Ulk1 (S555; 5869) and p44/42 MAPK (total-Erk1/2; 9102) were obtained from Cell Signaling Technology (Boston, MA, USA). The antibodies of LC3 (M152-3), PARP-1 (13371-1-AP) and p62 (18420-1-AP) for immunoprecipitation were purchased from Medical & Biological Laboratories (Naka-ku, Nagoya, Japan) and Proteintech (Wuhan, Hubei, China). The antibodies for *γ*-H2AX (S139; ab26350) were obtained from Abcam (Cambridge, MA, USA), and the antibody against actin (TA-09) was acquired from ZhongShanJinQiao Biocompany (Beijing, China). The antibody of p62 (sc-28359) for immunoblotting and immunofluorescence, the siRNA specific for human MAP LC3*β* (sc-43390), Ulk1 (sc-44182), p62 (sc-25575), Rad51 (sc-36361) and PARP-1 (sc-29437) were purchased from Santa Cruz Biotechnology (Dallas, TX, USA), along with the control siRNA (sc-37007). The siRNA targeted for Ulk1 (L-005049-00), MAP LC3*β* (L-012846-00) and Non-targeting Pool (D-001810-10-05) were also obtained from Dharmacon (Lafayette, CO, USA). Alexa Fluor 594 goat anti-rabbit IgG (H+L) (R37117) and Alexa Fluor 488 goat anti-mouse IgG (H+L) (A-11001) were purchased from Molecular Probes (Eugene, OR, USA). Rasfonin is stored in our lab.

### Cell culture and immunoblotting analysis

786-O, ACHN, HeLa, HepG2, HEK293T and K562 cells were grown in DMEM media containing 10% fetal bovine serum (GIBCO, Grand Island, NY, USA) and 1% antibiotics. Cells were grown to 70–80% confluency before addition of a variety of compounds. For siRNA interference, cells of 30% confluence in the media without antibiotics were transfected using DharmaFECT (Dharmacon, T2001) according to the manufacturer’s instructions. Cells were split and cultured overnight before stimulations after transfection for 48 h. Whole-cell lysates were prepared with lysis using Triton X-100/glycerol buffer, containing 50 mM Tris-HCl (pH 7.4), 4 mM EDTA, 2 mM EGTA and 1 mM dithiothreitol, supplemented with 1% Triton X-100, 1% SDS and protease inhibitors, and then separated on a SDS-PAGE gel and transferred to PVDF membrane. Immunoblotting was performed using appropriate primary antibodies and horseradish peroxidase-conjugated suitable secondary antibodies, followed by detection with enhanced chemiluminescence (Pierce Chemical, Rockford, IL, USA).

### Subcellular fractionation

Cells were seeded into 100 mm dishes at 90% confluency. After the indicated treatment, cells were gathered, pelleted by centrifugation at 3000 r.p.m. for 5 min, and washed three times with cold PBS. In all, 20% cells were resuspended in Triton X-100/glycerol buffer and labeled as the total homogenate. Method A: the other cells were resuspended in 400 *μ*l homogenization buffer A (10 mM Hepes-KOH (pH 7.9), 10 mM KCl, 1.5 mM MgCl_2_, 0.5 mM PMSF and 0.5 mM dithiothreitol) containing 0.5% NP-40, and then the homogenate was centrifuged at 3000 r.p.m. at 4 °C for 5 min after static on ice for 15 min. The supernatant was collected as the nuclear cytoplasm (Cyto). After washing twice with 400 *μ*l buffer A without NP-40, the pellet was resuspended in 60 *μ*l buffer C (20 mM Hepes-KOH (pH 7.9), 600 mM KCl, 1.5 mM MgCl_2_, 0.2 mM EDTA and 25% glycerol). After rotating on ice for 15 min, the homogenate was centrifuged at 13 000 r.p.m. at 4 °C for 15 min, and the supernatant was collected as the soluble nuclear fractions (Nu), and pellets were collected as the insoluble nuclear participates (Nup). After adding 30 *μ*l 3 × loading buffer or 60 *μ*l 1 × loading buffer to the Nu or Nup, respectively, the samples were boiled at 96 °C for 15 min before separating on a SDS-PAGE gel. Method B: the other cells were subjected to a nuclear extraction kit (Thermo Scientific, Waltham, MA, USA; 78835), and the Cyto and Nu fractions were extracted following the instructions. Immunoprecipitation was performed in the Cyto and Nu fractions extracted using Method B.

### Immunoprecipitation

Whole-cell lysates were prepared with lysis using Triton X-100/glycerol buffer as mentioned above. Rad51, p62 and pUlk1 were immunoprecipitated using the corresponding antibodies at 4 °C for 3 h, followed by 1-h incubation with Protein A-Sepharose (Vigorous Biotechnology, Beijing, China). Protein G-Sepharose (Vigorous Biotechnology) was performed using the antibody of LC3 to immunoprecipitate the protein. Immunoprecipitates and cell lysates were electrophoresed on SDS-PAGE, and subjected to immunoblotting analysis.

### Fluorescence microscopy

786-O cells were plated on glass cover slips and the indicated treatments were performed. Cells were washed with Ca^2+^- and Mg^2+^-free PBS (CMF-PBS), fixed with freshly prepared 4% paraformaldehyde at 4 °C for 30 min and permeabilized incubation with CMF-PBS containing 0.1% Triton X-100 and 0.5% BSA at room temperature (RT) for 5 min. Cells were then washed three times with CMF-PBS, blocked in CMF-PBS containing 3% BSA for 1 h, and incubated with the indicated antibodies in the presence of 0.1% Triton X-100 and 0.5% BSA. After washing three times, cells were incubated with the secondary antibodies diluted in CMF-PBS containing 0.5% BSA for 1 h. Cells were then immersed in VECTASHIELD with DAPI (H1200) to visualize the nuclei after washing three times. Images were acquired via Fluorescence microscopy (Zeiss, Heidenheim, Germany).

### Electron microscopy

Samples were washed three times with PBS, trypsinized and collected by centrifuging. Cell pellets were fixed with 4% paraformaldehyde at 4 °C overnight, post-fixed with 1% OsO_4_ in cacodylate buffer at RT for 1 h, and dehydrated stepwise with ethanol. The dehydrated pellets were rinsed with propylene oxide at RT for 30 min and embedded in Spurr resin for sectioning. Images of thin sections were observed under a transmission electron microscope (JEM1230, Akishima, Tokyo, Japan).

### RNA extraction and qPCR analysis

The total cellular RNA was extracted using TRIzol reagent (Invitrogen, Carlsbad, CA, USA; 15596-018) according to the manufacturer’s protocol, and 1 *μ*g of RNA was reversely transcribed at 42 °C for 60 min in 20 *μ*l PrimeScriptTM RT reagent Kit (TaKaRa, Dalian, Liaoning, China; DRR037A). Reactions were stopped by heat inactivation at 85 °C for 5 s. Primer sequences used for amplification were as follows:


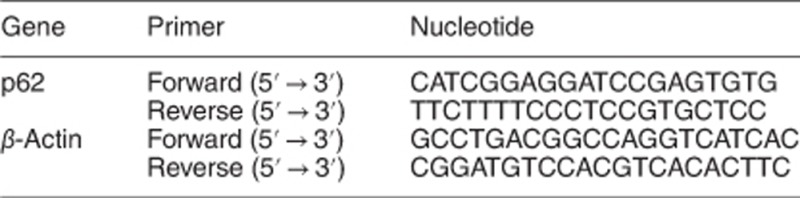


qPCR (CFX96; Bio-Rad, Hercules, CA, USA) was initiated with a 10-min denaturation at 95 °C in a final volume of 20 *μ*l. The cycle profile was 95 °C (15 s), 60 °C (45 s) and 72 °C (1 min) for up to 40 cycles. The data were calculated based on the internal control of *β*-actin.

### Statistical analysis

The images were analyzed by Image J (National Institutes of Health, Bethesda, MD, USA) to verify the linear range of chemiluminescence signals and quantifications were carried out using densitometry, and mean±S.D. were shown in histograms along with the blots. The normally distributed data are shown as mean±S.D. and analyzed using one-way analysis of variance and the Student–Newman–Keuls post-hoc test. For the non-normally distributed data in the electron microscopy, results were show as mean, and analyzed using Friedman test.
