# Supplementary figures and images for: Sunitinib induces genomic instability of renal carcinoma cells through affecting the interaction of LC3-II and PARP-1
Source: Cell Death Dis. 2017 Aug 10;8(8):e2988–. doi: 10.1038/cddis.2017.387 (PMC5596573; doi:10.1038/cddis.2017.387)

Figure 1

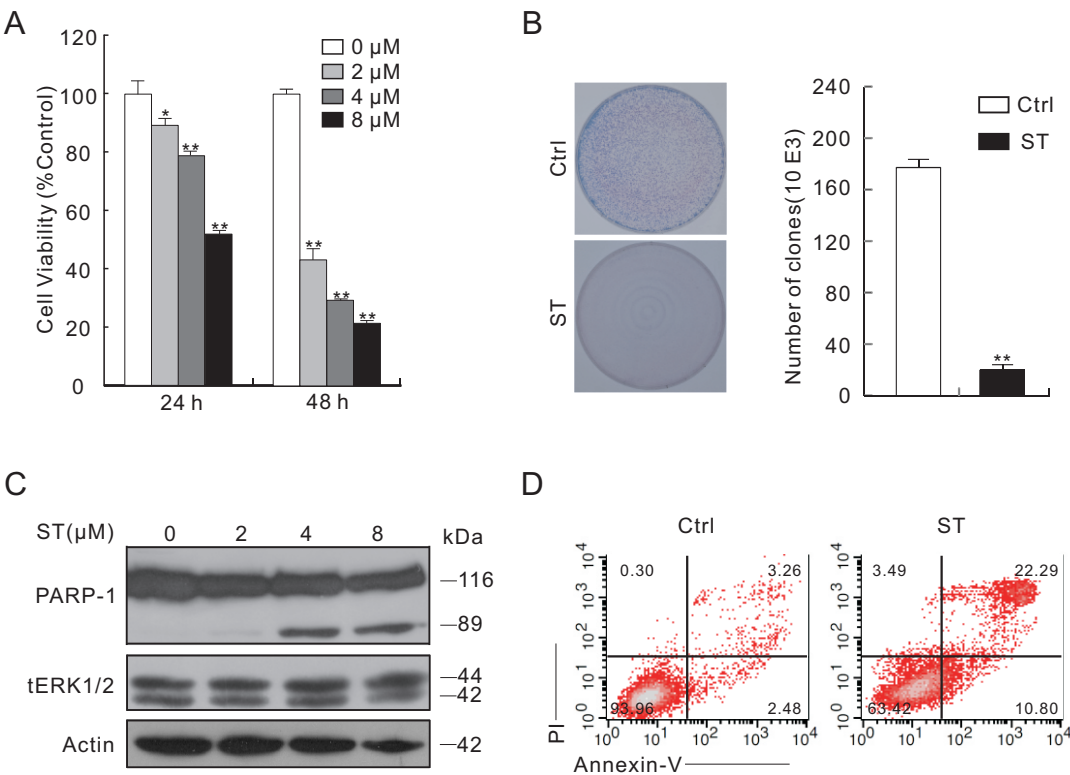

Supplement: Supplementary Figure 1 [file cddis2017387x1.pdf]

Figure 2

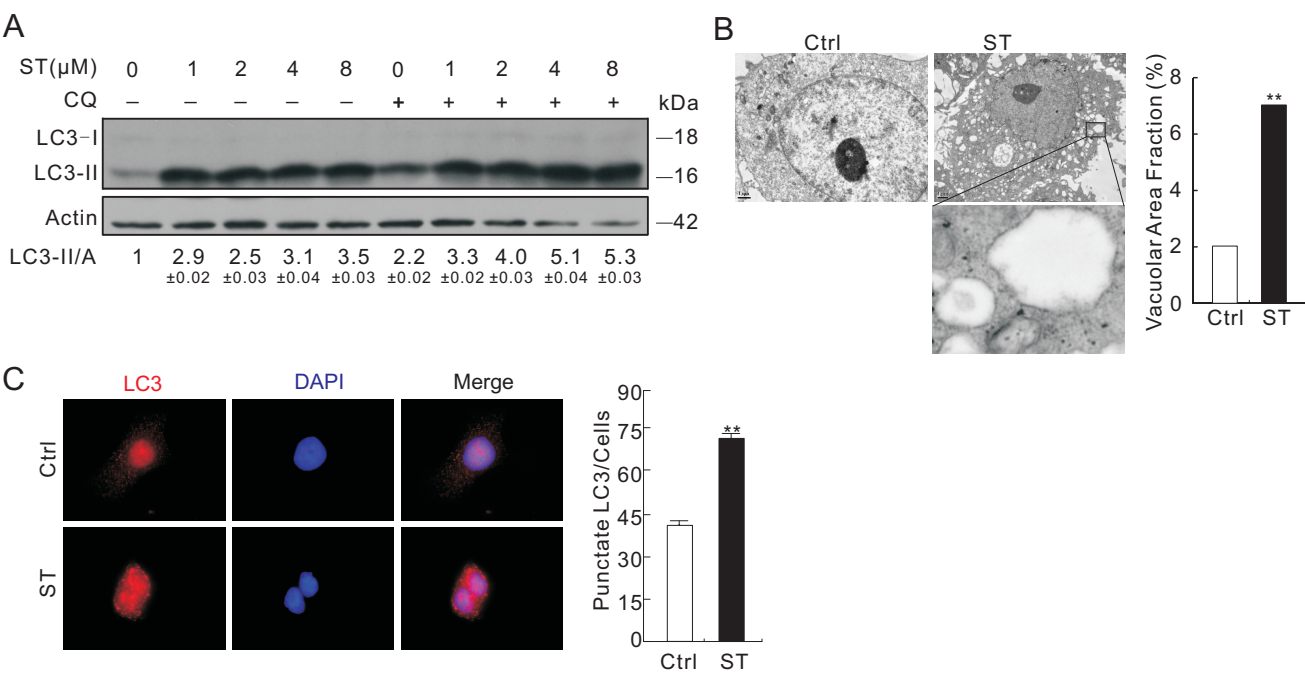

Supplement: Supplementary Figure 2 [file cddis2017387x2.pdf]

Figure 3

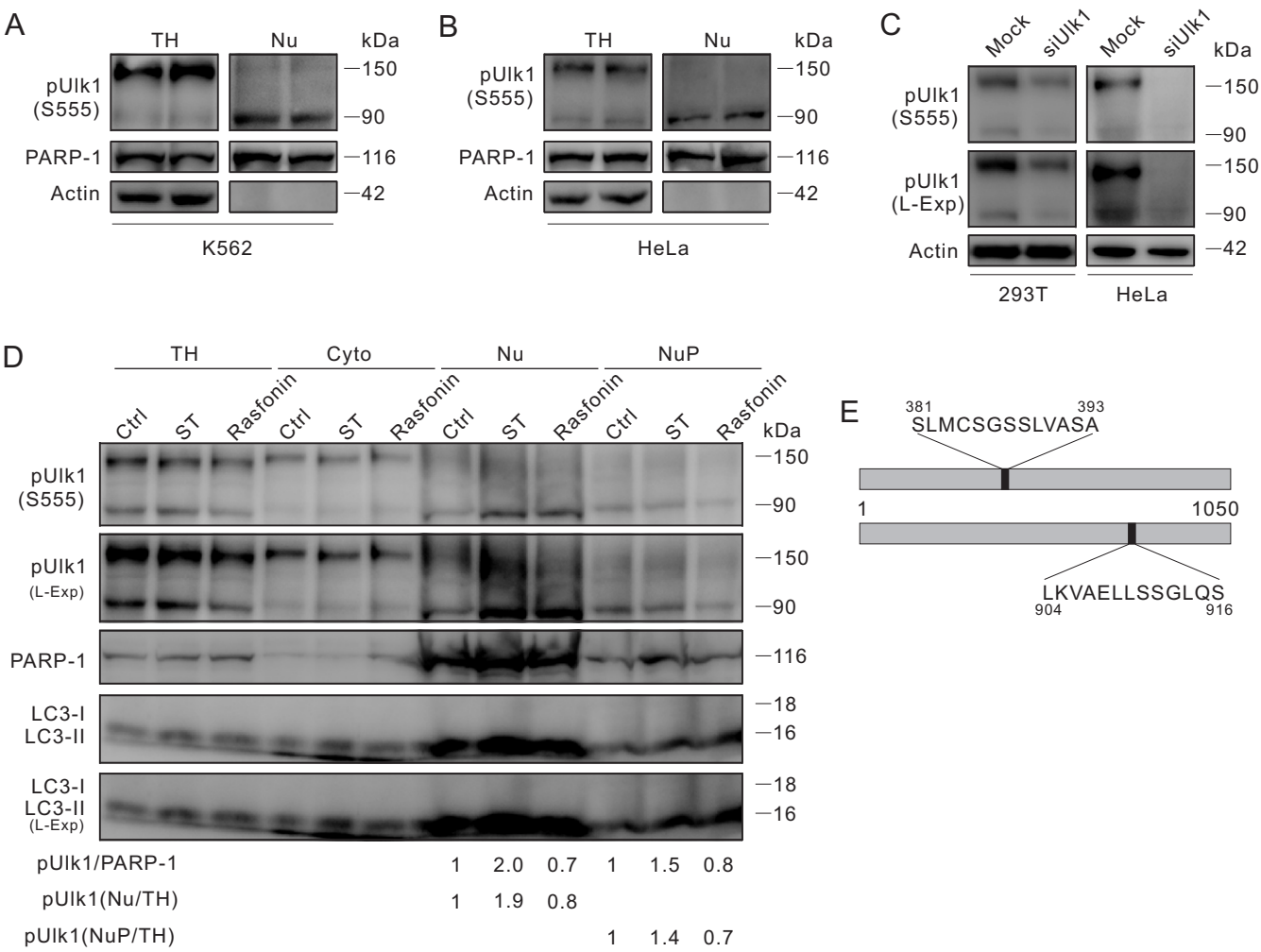

Supplement: Supplementary Figure 3 [file cddis2017387x3.pdf]

Figure 4

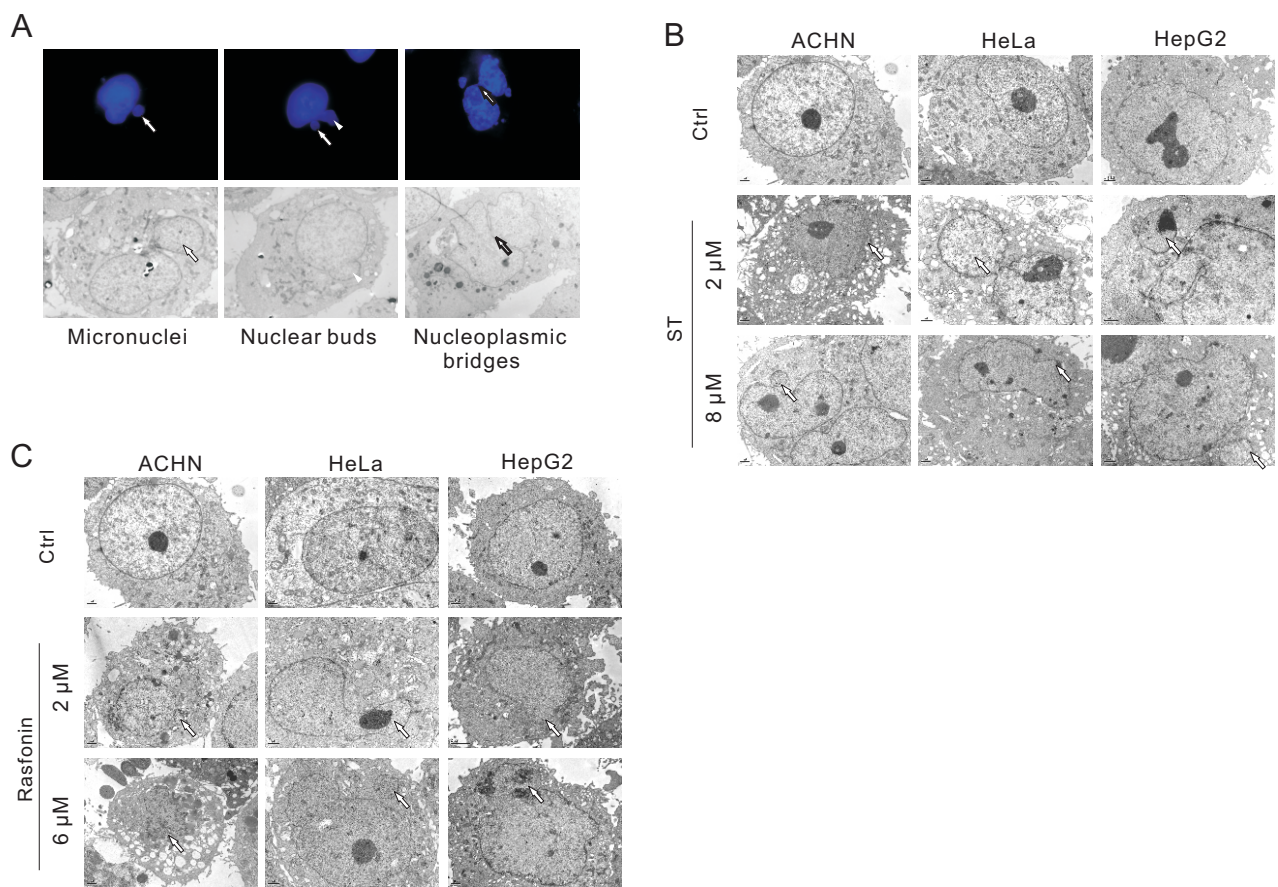

Supplement: Supplementary Figure 4 [file cddis2017387x4.pdf]

Figure 5

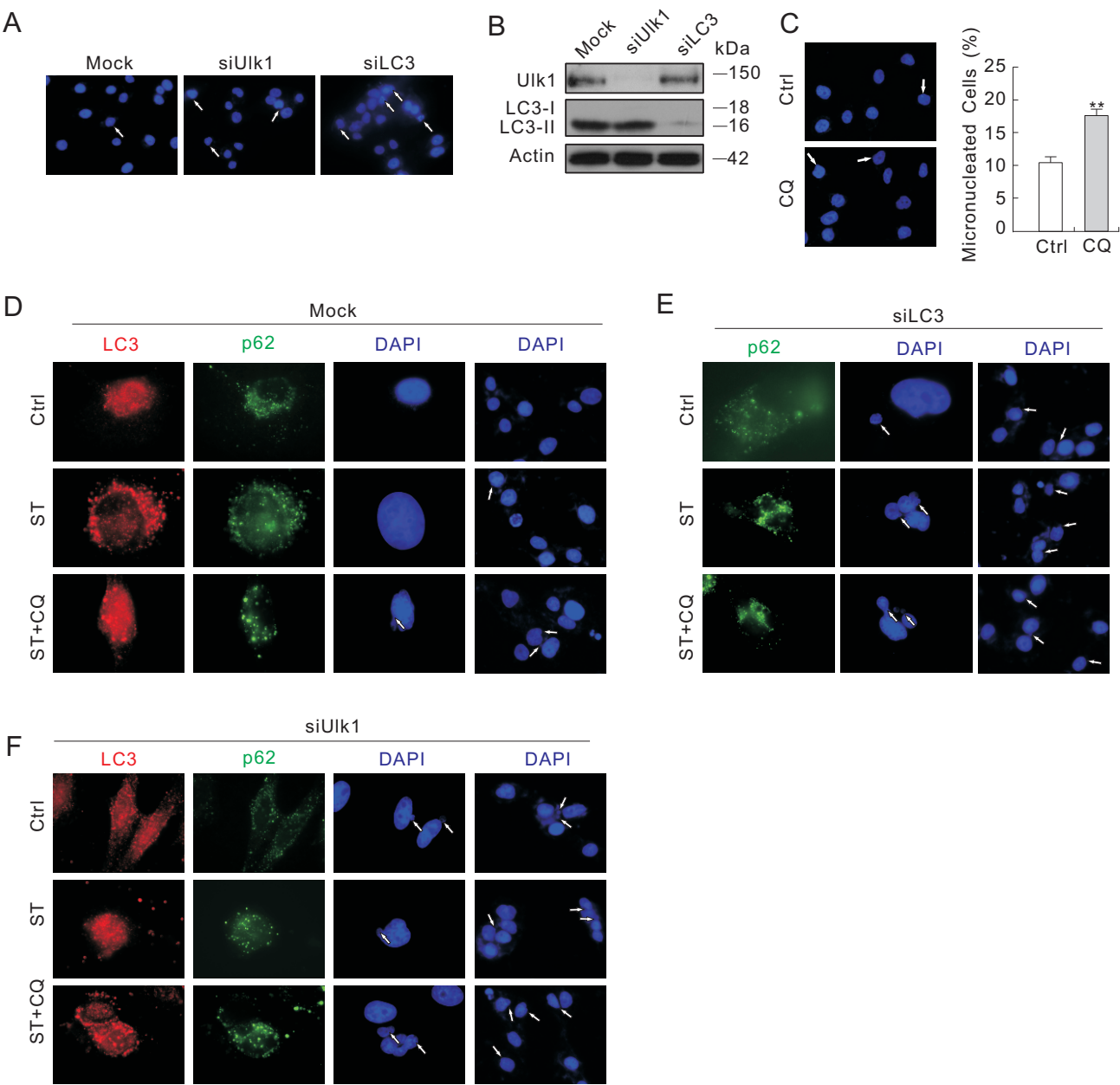

Supplement: Supplementary Figure 5 [file cddis2017387x5.pdf]

Figure 6

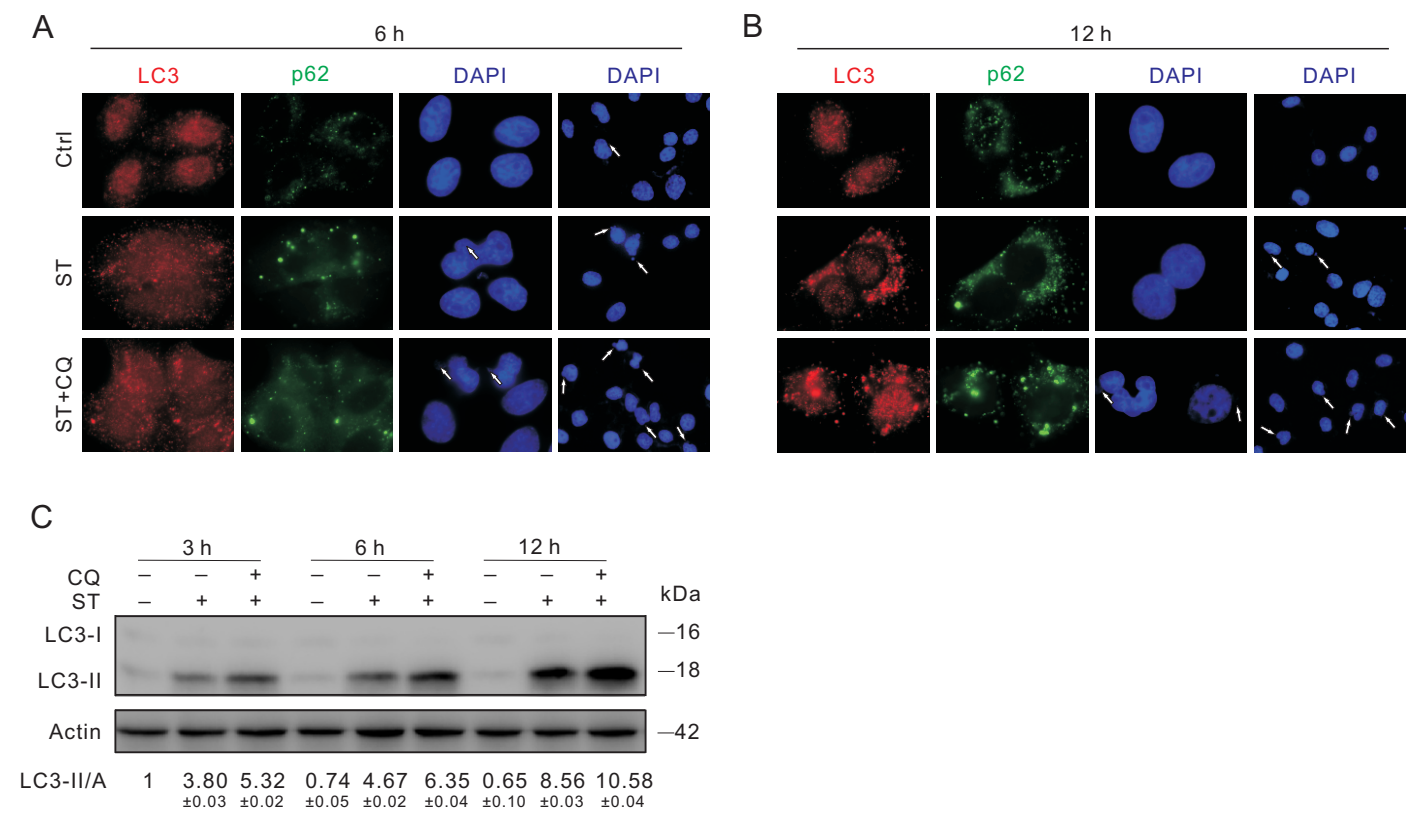

Supplement: Supplementary Figure 6 [file cddis2017387x6.pdf]

Figure 7

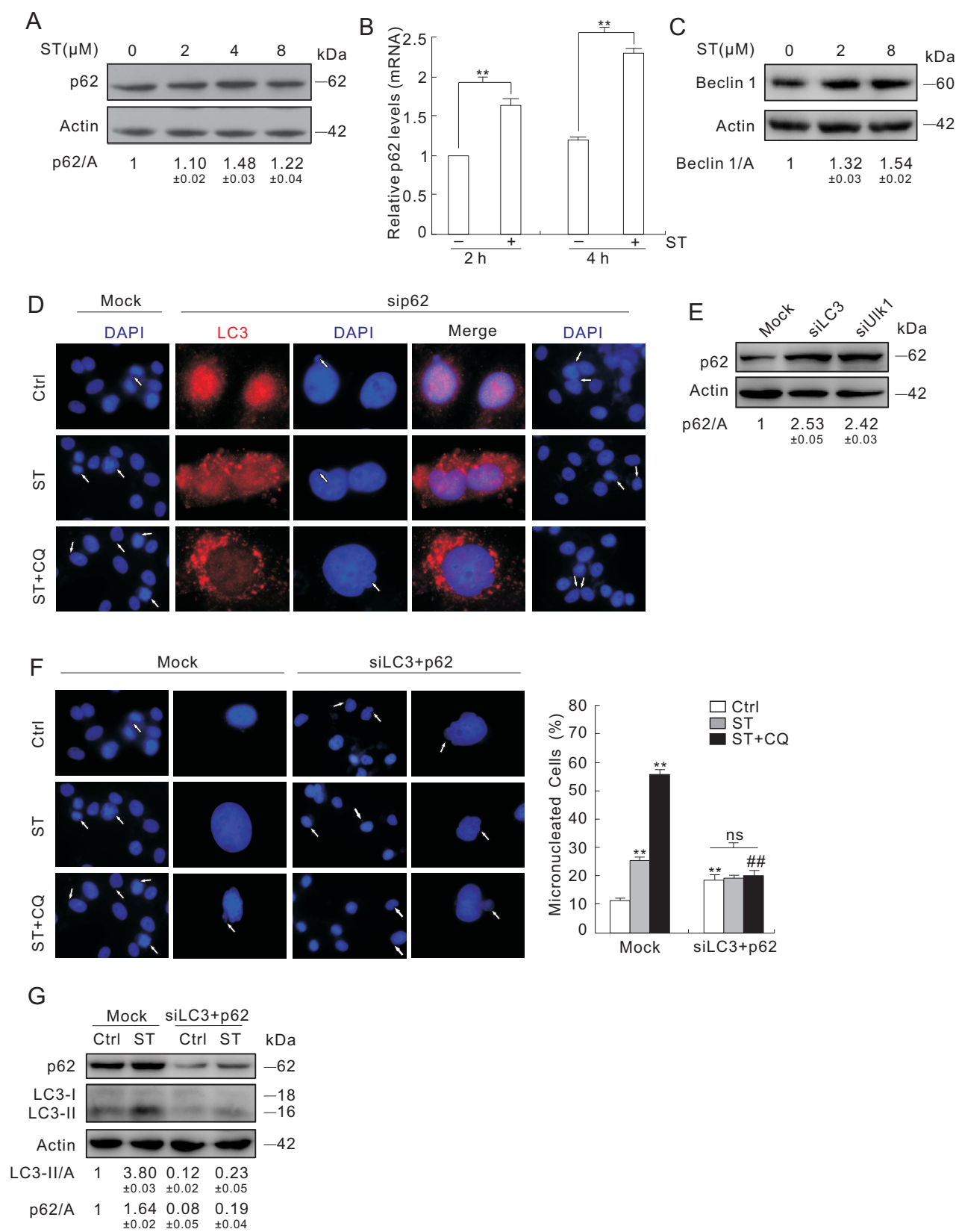

Supplement: Supplementary Figure 7 [file cddis2017387x7.pdf]

Figure 8

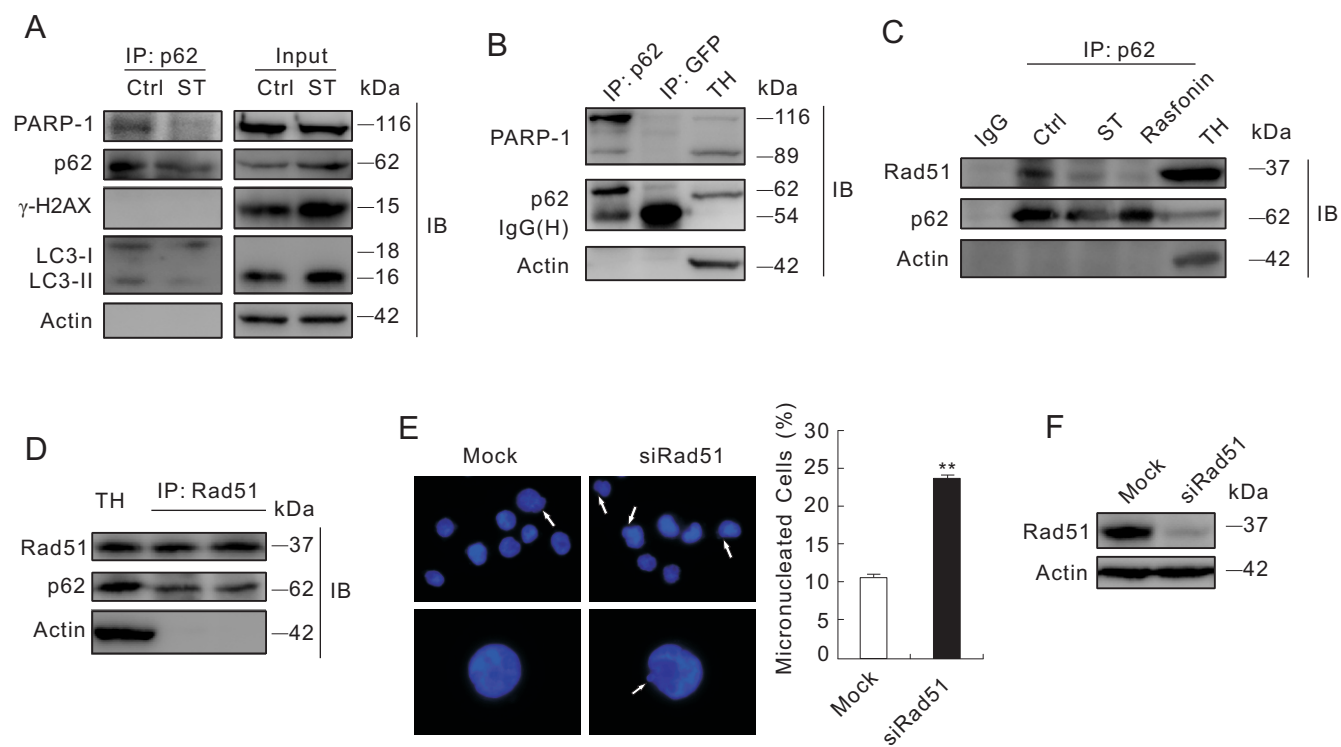

Supplement: Supplementary Figure 8 [file cddis2017387x8.pdf]

Figure 9

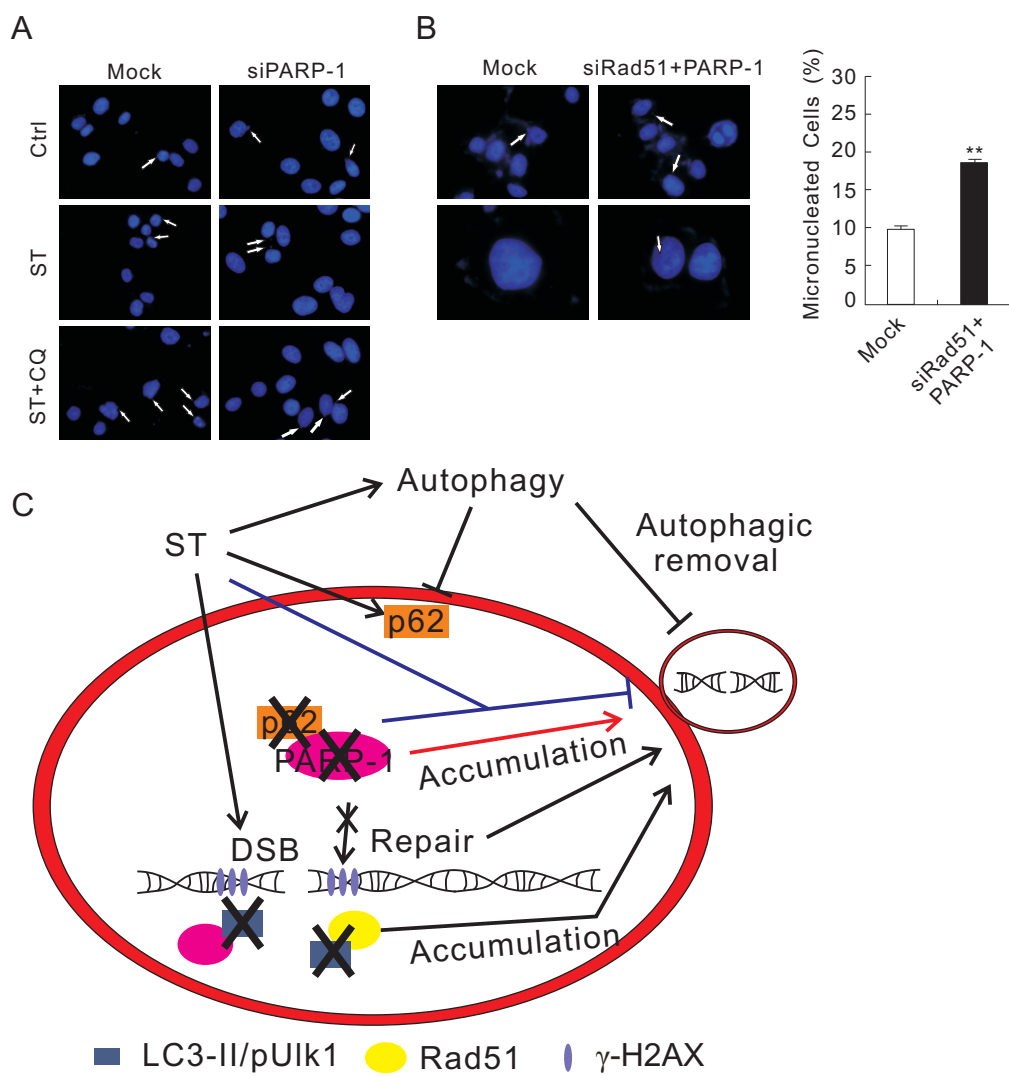

Supplement: Supplementary Figure 9 [file cddis2017387x9.pdf]
