# Supplementary material for: Sunitinib induces genomic instability of renal carcinoma cells through affecting the interaction of LC3-II and PARP-1
Source: Cell Death Dis. 2017 Aug 10;8(8):e2988–. doi: 10.1038/cddis.2017.387 (PMC5596573; doi:10.1038/cddis.2017.387)
Supplement: Supplementary Figure Legends [file cddis2017387x10.docx]

**Sunitinib induces genomic instability of renal carcinoma cells through affecting the interaction of LC3-II and PARP-1**

**Supplementary Figure legends**

**Figure 1. ST inhibits cell viability and induces apoptosis.** (A) 786-O cells were treated with ST (0–8 μM) for 24 h, and cell viability was analyzed by MTS assay as described in ‘Materials and methods’. (B) Colony growth assays were performed using 2 μM ST, and the number of colony were shown in graph. **P < 0.01 vs. control. (C) 786-O cells were treated with ST (0-8 μM) for 12 h, and the cell lysates were prepared and analyzed by immunoblotting using the indicated antibodies. (D) Following treatment with 8 μM ST for 12 h, 786-O cells were stained and monitored by flow cytometry. Similarly experiments were repeated for at least three times.

**Figure 2. ST induces autophagy in ACHN cells.** (**A**) Following treatment of ST (8 μM; unless otherwise indicated) for 3 h in the presence or absence of CQ (10 μM), ACHN cells were lysed and subjected to immunoblotting with the antibodies indicated. (**B**) Electron microscopy was performed for ACHN cells following treatment with ST for 3 h, and the data of the vacuolar area fraction were calculated for at least 15 cells and shown in graph. (**C**) Immunofluorescence using LC3 antibody was performed for ACHN cells following treatment with ST for 3 h, the number of the punctate LC3 in each cell was counted, and at least 50 cells were included for each group. The Data representing the mean ± S.D. were shown in graph. **P < 0.01 vs. control. The data represent three independent experiments.

**Figure 3. Nuclear localization of LC3-II and pUlk1.** (**A** and **B**) TH and Nu were extracted from K562 or HeLa cells. (**C**) Following transfection with Ulk1 or the Mock control siRNAs for 48 h, HEK293T and HeLa cells were lysed and subjected to immunoblotting with the antibodies indicated. (**D**) TH, Cyto, Nu, and the Nup (insoluble nuclear participates) were extracted from HEK293T cells after treatment with the indicated compounds for 3 h, and the fractions were analyzed by immunoblotting with the antibodies indicated. (**E**) Schematic diagram of homo species Ulk1 and the predicated cleavage sites. Similarly experiments were carried out for at least three times.

**Figure 4. ST and rasfonin increase the frequency of micronuclei in a variety of cell lines.** (**A**) Three types of genotoxic biomarkers were observed in the ST-treated 786-O cells (arrow: micronuclei; arrowhead: nuclear buds; bold arrow: nucleoplamic bridges). (**B** and **C**) Electron microscopy was performed for ACHN, HeLa, and HepG2 cells after treatment with either (**B**) ST (0–8 μM) or (**C**) rasfonin (0–6 μM) for 3 h. Representative images were shown with the arrows indicating micronuclei. Similarly experiments were carried out for at least three times.

**Figure 5. Inhibition of autophagy blocks the removal of micronuclei.** (**A** and **B**) After transfection with siRNA from Dharmacon target Ulk1 or LC3 for 48 h, images of 786-O cells were obtained using fluorescence microscopy after labeling with DAPI (**A**), cell lysates were analyzed by immunoblotting with the indicated antibodies (**B**). Representative images were presented and the white arrows indicated micronuclei. (**C**) 786-O cells were treated with or without CQ for 12 h, images were obtained by fluorescence microscopy, and the percentages of micronuclei were calculated and presented as histogram graph (the arrows indicate micronuclei). (**D**-**F**) 786-O cells were transfected with siRNA target Ulk1 or LC3 for 48 h, and treated with ST or a combination with CQ for 12 h, and the images were obtained using fluorescence microscopy after labeling the antibodies of LC3 and p62 (400 and 1000 magnification). The images shown here are the separate channels of Figure 6**c**. The data represent three independent experiments.

**Figure 6. The percentage of micronuclei varies with the period of ST-treatment.** (**A** and **B**) 786-O cells were treated with ST or a combination with CQ for up to 12 h, immunofluorescence was performed, and the images were obtained using fluorescence microscopy after labeling the antibodies of LC3 and p62 (400 and 1000 magnifications). The images shown here are the separate channels of Figure 6**e**. (**C**) Cell lysates were prepared after indicated treatment and analyzed by immunoblotting using the indicated antibodies. At least three independent experiments were performed.

**Figure 7. ST fails to increase the formation of micronuclei in the p62-depleted cells.** (**A**) Following treatment with ST for 3 h in the presence or absence of CQ, ACHN cells were lysed and subjected to immunoblotting with the antibodies indicated. (**B**) qPCR was performed to detect the mRNA expression of p62 following treatment of ST for up to 4 h in 786-O cells. (**C**) Following treatment with ST for 3 h, 786-O cells were lysed and subjected to immunoblotting with the antibodies indicated. (**D**) 786-O cells were transfected with siRNA target p62 for 48 h, treated with ST or a combination with CQ for 12 h, and images were obtained using fluorescence microscopy after labeling the antibodies of LC3 and p62. The images shown here are the separate channels of Figure 7C. (**E**-**G**) Following transfection with the indicated siRNAs for 48 h, 786-O cells were treated with ST in the presence or absence of CQ for 12 h, cell lysate were prepared and immunoblotting was performed with the antibodies indicated (**E** and **G**); Images were obtained using fluorescence microscopy with 400 and 1000 magnifications, and the arrows indicate micronuclei (**F**). **P (##P) < 0.01 vs. control. At least three independent experiments were performed.

**Figure 8. Rad51 interacts with p62 to affect the formation of micronuclei in HeLa cells.** (**A**) After ST treatment for 3 h, immunoprecipitation was performed for 786-O cells with the antibody against p62. (**B**) Immunoprecipitation was performed for HEK293T cells with the antibodies against p62 or GFP. (**C**) HEK293T cells were incubated with ST or rasfonin for 3 h and lysed, and p62s were precipitated using the antibody against p62. (**D**) Immunoprecipitation was performed for HeLa cells with the antibodies against Rad51, and the immunoprecipitates were resolved by electrophoresis and probed by immunoblotting with the indicated antibodies. (**E** and **F**) Following transfection with the Rad51 siRNAs for 48 h, HeLa cells were stained with DAPI. Images were obtained by fluorescence microscopy with 400 and 1000 magnifications, the arrows indicated micronuclei and the histogram graph data representing the mean ± S.D. were shown (**E**), **P < 0.01 vs. control. Cell lysate were prepared and immunoblotting was performed with the indicated antibodies (**F**). Similarly experiments were carried out for at least three times.

**Figure 9. Depletion of PARP-1 significantly increases the frequency of micronuclei.** (**A**) After transfection with PARP-1 siRNA for 48 h, 786-O cells were incubated with ST for 12 h, images were obtained using fluorescence microscopy with 400 magnification. (**B**) Following transfection with Rad51 and PARP-1 siRNAs for 48 h, 786-O cells were stained with DAPI. Images were obtained by fluorescence microscopy with 400 and 1000 magnifications, the arrows indicated micronuclei, and the histogram graph data representing the mean ± S.D. were shown. **P < 0.01 vs. control. The data represent three independent experiments. (**C**) Schematic mechanism of the autophagy related protein in ST-induced nuclear instability.
